# Hospital Drains as Reservoirs of *Pseudomonas aeruginosa*: Multiple-Locus Variable-Number of Tandem Repeats Analysis Genotypes Recovered from Faucets, Sink Surfaces and Patients

**DOI:** 10.3390/pathogens6030036

**Published:** 2017-08-09

**Authors:** Cindy Lalancette, Dominique Charron, Céline Laferrière, Patrick Dolcé, Eric Déziel, Michèle Prévost, Emilie Bédard

**Affiliations:** 1 INRS-Institut Armand-Frappier, Laval, QC H7V 1B7, Canada; cindy.lalancette@inspq.qc.ca (C.L.); eric.deziel@iaf.inrs.ca (E.D.); 2 Department of Civil Engineering, Polytechnique Montréal, Montréal, QC H3T 1J4, Canada; dominique.charron@polymtl.ca (D.C.); michele.prevost@polymtl.ca (M.P.); 3 Department of microbiology, infectious diseases and immunology, Université de Montréal, Montréal, QC H3T 1J4, Canada; lafischer@live.ca; 4 Department of Medical Microbiology and Infectious Diseases, Centre Hospitalier Régional de Rimouski, Rimouski, QC G5L 5T1, Canada; patrick.dolce.crsssr@ssss.gouv.qc.ca

**Keywords:** sink environment, MLVA, genotyping, environmental reservoir, *Pseudomonas aeruginosa*, heathcare-acquired infections

## Abstract

Identifying environmental sources of *Pseudomonas aeruginosa* (*Pa*) related to hospital-acquired infections represents a key challenge for public health. Biofilms in water systems offer protection and favorable growth conditions, and are prime reservoirs of microorganisms. A comparative genotyping survey assessing the relationship between *Pa* strains recovered in hospital sink biofilm and isolated in clinical specimens was conducted. Environmental strains from drain, faucet and sink-surface biofilm were recovered by a culture method after an incubation time ranging from 48 to 240 h. The genotyping of 38 environmental and 32 clinical isolates was performed using a multiple-locus variable-number of tandem repeats analysis (MLVA). More than one-third of *Pa* isolates were only cultivable following ≥48 h of incubation, and were predominantly from faucet and sink-surface biofilms. In total, 41/70 strains were grouped within eight genotypes (A to H). Genotype B grouped a clinical and an environmental strain isolated in the same ward, 5 months apart, suggesting this genotype could thrive in both contexts. Genotype E grouped environmental isolates that were highly prevalent throughout the hospital and that required a longer incubation time. The results from the multi-hospital follow-up study support the drain as an important reservoir of *Pa* dissemination to faucets, sink surfaces and patients. Optimizing the recovery of environmental strains will strengthen epidemiological investigations, facilitate pathway identification, and assist in identifying and controlling the reservoirs potentially associated to hospital-acquired infections.

## 1. Introduction

Healthcare-associated infections represent an important burden worldwide [1]. *Pseudomonas aeruginosa* (*Pa*) is recognized as a common cause of healthcare-associated infections, and has been considered an important causative agent in hospital outbreaks over the last decade [2,3,4]. A large number of these outbreaks have been linked to environmental sources, especially the water system [5,6,7,8,9]. Faucets, associated components, drains and connection plumbing can function as reservoirs for *Pa*, especially in the presence of microbial biofilms, which can harbor microorganism communities, protecting them from environmental stresses and favoring their growth [10]. Indeed, prospective studies have established that up to 100% of faucets [11,12,13] and drains [14,15,16] in hospitals are contaminated with *Pa*. However, the identification of the environmental sources associated to opportunistic pathogen infections represents an important and growing challenge. This is especially critical in a context for which patients have increased clinical burden and where sources of nosocomial infections must be identified, recognized and controlled.

To assess the presence of *Pa* in the hospital environment, most epidemiological investigations rely on culture-based methods developed for medical clinical microbiology [9,12,17]. Culture methods are so far considered an essential first step to recover strains that can undergo genotyping, and should therefore aim to recover all environmental strains to improve the likelihood of recovering those responsible for the outbreaks. Various growth media can be used for *Pa* isolation, including commonly used cetrimide agar plates [5,13,18,19] and *Pa* selective agar [20,21,22,23]. However, the culture media and conditions optimized for clinical strains may not be optimal for the recovery, isolation and quantification of environmental strains that are often adapted to lower nutrient levels and temperatures, typically associated to biofilms. Quantification using nutrient-poor growth media and incubation at lower temperatures (25–30 °C vs 37–41 °C) over a longer period of time (7 days vs 24–48 h) have been suggested to maximize the bacterial recovery from environmental samples [24].

Previous studies suggest multiple environmental reservoirs [9,11,16,25], pathways of dissemination [15,26,27] and sources of contamination for *Pa* [12,28,29,30], especially within the sink environment. In outbreak situations, genotyping methods are used to establish whether one of the strains isolated from the environment is a possible source of clinical infection. Several methods have been reported in clinical genotyping studies, including pulsed-field gel electrophoresis (PFGE), multiple-locus variable-number of tandem repeats analysis (MLVA), multilocus sequence typing (MLST), and repetitive element sequence-based polymerase chain reaction (rep-PCR). Given the large number of strains that could be present in the sink environment within hospitals, the selected method should have a high level of discrimination while requiring low labor and cost. For these reasons and on the basis of results obtained by van Mansfeld et al., who compared PFGE, MLST and MLVA methods to investigate the population structure of 60 strains isolated from cystic fibrosis patients [31], the MLVA method was selected to perform the genotyping study between the environmental and clinical strains. MLVA is a high-resolution and easy-to-perform method [32] that is based on the analysis of the selected variable-number of tandem repeats (VNTR) amplified by PCR and detected by electrophoresis. Its high discriminatory power makes it useful for outbreaks or short-term investigations [31,33,34,35]. The number of VNTR loci selected for the analysis can vary [34,36,37], and the probability of associating two unrelated strains to a same genotype is evaluated using the combined Hunter–Gaston diversity index (HGDI) [37].

Increased knowledge of the environmental reservoirs and pathways of dissemination of *Pa* within healthcare facilities is intensely needed to better control the bacterial load and exposure, and therefore hope to reduce the risk of infection. The objectives of the present study were to: (1) compare the culture media and incubation time to improve the recovery of environmental *Pa* strains from water and biofilms in sink components, (2) link isolated environmental and clinical specimens through genotypic analysis, and (3) understand faucet and drain environmental colonization patterns and interrelationships through a multi-hospital occurrence study.

## 2. Results

### 2.1. Culture Protocol Comparison for Environmental Samples

A total of 47 phenotypically different colonies of presumed *Pa* were isolated from 21/57 swab samples collected in hospital A, either on acetamide agar (Aa), on *Pseudomonas* isolation agar (PIa) or on both media over the various studied incubation periods (Table 1). The non-contaminated sinks were distributed throughout various floors and wards, and had no observed patterns or trends compared to the positive sinks. Confirmation by the International Organization for Standardization culture method (ISO 16266) and by PCR (*gyrB*) was obtained for 38/47 isolates. The 38 isolates were recovered from 17 *Pa*-positive swab samples (Table 1), collected in 8 of the 18 sampled sink environments. While a comparable number of isolates were recovered from the drains by Aa and PIa, the number of isolates from the faucet and sink-surface swabs was strikingly higher on PIa. Drain and faucet swabs had a high rate of confirmation on PIa, despite the extensive incubation time required in some cases; only one isolate from a faucet swab was not confirmed as *Pa*. When considering both culture media tested, 37.5% of strains grew after 48 h of incubation and 17.5% grew after 10 days. Positivity was found to be significantly lower for faucets and sink surfaces than for drains when considering the culture results obtained after 48 h of incubation (Table 2). Conversely, *Pa* positivity was not statistically different between drains, faucets and sink surfaces when accounting for positive samples detected after up to 10 day incubation time (Table 2).

### 2.2. Genotyping of Environmental and Clinical Strains

**Figure 1:**
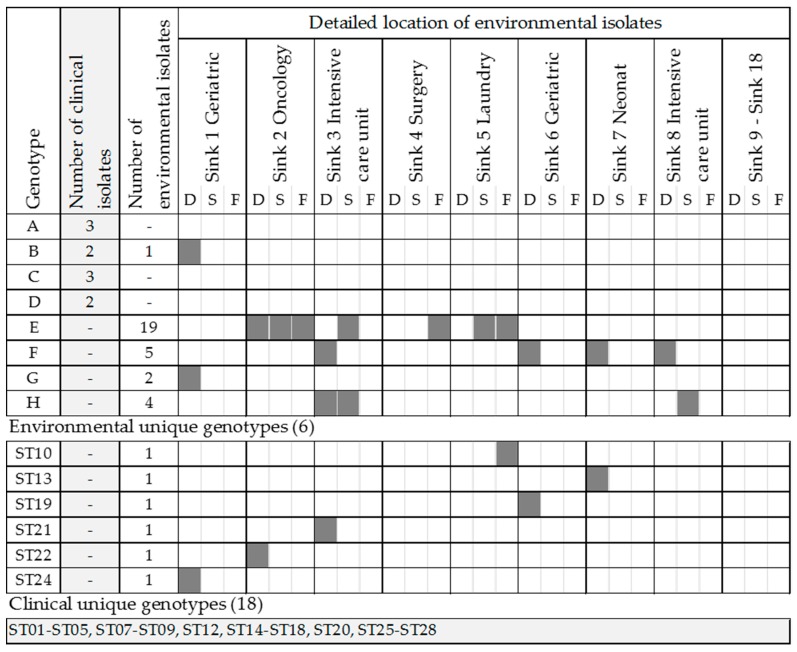
Distribution of clinical and environmental isolates per genotype and detailed location of environmental isolates per site of isolation: drain (D), sink surface (S) or faucet (F).

The genotyping of 38 *Pa* environmental isolates and 32 clinical isolates collected from hospital A was performed using MLVA with seven loci (MLVA-7). The number of alleles per locus varied between one and nine. MLVA-7 analyses resulted in eight genotypes grouping 41 strains, 27 single-strain genotypes and 3 not-classified strains with less than 5 VNTR amplified (Figure 1; Table S1). One locus could not be amplified for 10 environmental and 6 clinical strains, and at least two loci could not be amplified for 1 environmental and 9 clinical strains, resulting in an overall typability of between 82% and 96% for all strains, and a HGDI of between 0.49 and 0.83 (Table 3). Of the 18 sinks sampled, 8 had at least one positive site with a *Pa* strain from genotypes B and E to H (Figure 1). When available, the location of the suspected source of infection was documented for clinical isolates (Table S1).

### 2.3. Occurrence and Relationship between Sink Partsthis is an Example of an Equation

The results from the occurrence study conducted in five hospitals (A to E) revealed that 50.1% of all sampled sink drains were *Pa* culture-positive, whereas surfaces, faucet swabs and water had lower positivity (12.7%, 2.6% and 4.3%, respectively). The percentage of the culture positivity of sampled sites from hospitals B to E were compatible with the results from hospital A obtained after 48 h of incubation (Table 2). The paired positivity of sampling sites within a sink was evaluated for drains, faucets and sink surface swabs from hospitals A and B, and for water, faucets and drain swabs from hospitals B to E (Table 4). The odds were the highest for having a positive surface or water when the faucet swab was positive. However, because of the low number of positive faucets, increased odds of a positive faucet or surface in the presence of a positive drain should also be considered. Of all 229 sinks sampled, only 2 had positive results for all the sampled sites at those sinks.

## 3. Discussion

Nutrient-rich culture media with an antibiotic (PIa) and nutrient-poor selective media (Aa) were tested to assess the recovery of environmental strains over a prolonged incubation time. The rates of *Pa* isolation from drains were similar on PIa versus on Aa, whereas all but one isolate recovered from faucet and sink surface swabs were isolated on PIa (Table 1). The higher *Pa* recovery by PIa for faucet and sink surface swab samples could be associated to its nutrient-rich composition, most likely because it provided the necessary elements for the revival of cells under environmental stress or into latent state. The presence of viable but not culturable cells in the biofilm may have also contributed to the lack of culture positivity as well as the length of incubation time needed for the faucet and sink-surface swab samples [38].

The results suggest the importance of a longer incubation period for a better recovery of environmental isolates. More than one-third of the isolates required over 48 h of incubation, and half of those required as many as 10 days. When conducting an environmental investigation for water and swab samples, the choice of culture method and associated incubation times are key factors to improve the recovery of environmental isolates. Using the standard ISO culture method (48 h of incubation), a large proportion of positive sites may not be detected, compared to using other culture protocols with longer incubation times, such as the standard test method ASTM D5246-15 [39]. For example, the environmental strain that was genotypically similar to the clinical strain (genotype B) was isolated after 72 h of incubation time. Furthermore, some genotypes may require a longer incubation time than others. Although current results do not provide sufficient data to conclude, 18 of the 19 isolates recovered after 10 days of incubation belonged to the same genotype (E). The remaining isolate (Table S1: strain ID CL547b, genotype ST10) was closely related to genotype E, with one allele difference for the VNTR MS223, and was isolated from the same swab sample as CL547a (genotype E). Importantly, a longer incubation time did not reduce the rate of confirmation of isolated strains. All isolates recovered after a 10 day incubation time had a 100% confirmation by PCR and ISO culture methods, whereas strains recovered after 48 h had a lower confirmation rate of 78%.

Polymorphic VNTR were selected on the basis of the reported typability, the HGDI and the length, with a target of >100 bp. Longer VNTR have been reported to be more stable over time [32], and distinct alleles with longer repeat units are generally easier to score on agarose gels [40]. Provided that the clinical strains were isolated prior to the environmental isolates, a short, highly variable repeat unit would make it difficult to find a possible association between clinical and environmental strains. The 32 clinical isolates were grouped into 22 genotypes (18 unique), whereas the 38 environmental isolates were grouped into 11 genotypes (6 unique), displaying less diversity than clinical isolates previously observed [12]. Environmental isolates also showed a higher typability when averaging all seven VNTR (95 vs 82%). For example, the VNTR MS215 was not amplified from eight clinical strains (Table S1), resulting in a lower typability of 74% compared to a 100% typability for environmental strains. The lack of amplification for 2% of the tested isolates was previously reported for MS215 [31]. The lack of amplification of MS222 for all genotype C and F strains, and of MS223 for genotype H strains, suggested either the absence of those VNTR or mutation in the primer annealing site [27]. The two other strains that were missing MS222 were categorized as of unique genotypes (ST19 and ST20) but only displayed a difference in the number of repeats for MS142 to be part of genotype B (ST20) or F (ST19). Interestingly, ST19 was isolated from the same drain biofilm as two strains from genotype F (CL511 and CL512; Table S1), suggesting a possible change of the strain genotype over time.

The sink located in the intensive care unit (sink 3) had the most diversity in genotypes, with three genotypes isolated from the drain and two from the sink surface. Davis et al. also observed a larger diversity of *Pa* strains in neonatal ICUs during an outbreak investigation associated to sink transmission [29]. The presence of the same genotype both in the drain and on the surface area suggested splashing from the drain to the surface of sink 3 during water utilization, as previously reported [26]. Similarly, strains from the same genotype were recovered from the drain, the faucet and the sink surface in sink 2, located in Oncology. Overall, clinical and environmental isolates were not frequently grouped in the same genotypes, as previously observed [12,29]. In this study, only genotype B isolates were recovered from patients and sinks: from the drain in sink 1, located in the geriatric ward, and from two patients, a cystic fibrosis patient hospitalized 13 months earlier (undetermined site of acquisition) and a patient from the geriatric ward, 5 months after the environmental sampling. Both clinical and environmental isolates were small non-mucoid colonies with a metallic sheen. The MLVA-7 profile of genotype B strains was also identical to clinical strains isolated from two CF patients in Sweden [27]. When comparing clinical strains to previously published MLVA-7 profiles, genotype ST08 was found to be identical to a strain previously isolated both in hospital water and in patients, and identified as the source of an outbreak [27]. The isolation of the same genotype from both clinical and environmental samples raises questions with regard to the dissemination pathway and its chronology: from the patient to the sink drain, with a clinically fit strain able to colonize drain and water biofilms, or from the sink drain to the patient, with an environmentally fit strain able to successfully infect patients. A combination of both is also possible, where an environment is contaminated by patients and, in turn, becomes a source of contamination via splashing the hands of the caregiver [15,41], the aerator [28], the medical material used or stored near the sink, and the patient bed [26,42,43]. However, during an investigation, the exact location of *Pa* acquisition is not always clear, and sampling of the environment often occurs up to several weeks after acquisition, making it difficult to identify the source. Furthermore, the low culturability of *Pa* in the presence of environmental stressors increases the challenge of isolating all strains present within the sink environment. As molecular methods such as whole genome sequencing become more affordable and accessible to clinical settings, better identification of the source might be achieved [29,30]. Indeed, the study by Quick et al. revealed five clades to which clinical and environmental strains were closely related in a burn center [30]. In addition, the weekly sampling of the patient’s environment and water during their stay increased the likelihood of isolating environmental strains associated to the patient strains. In the present study, each of the sink environments were sampled only once.

The high prevalence of *Pa* in sampled sink drains (49.8%) was in line with results from previous studies summarized in a recent review [44]. Despite the high positivity of drains, a low positivity was detected for faucets (2.6%) and water (4.3%), suggesting that these environments were not as favorable for *Pa* growth or recovery because of conditions present at the faucet: shear forces due to water flow, cleaning and disinfection procedures, lower nutrient levels, exposure to chlorine, and metal aerators. As previously reported, such environmental stressors can impact the culturability and growth [45,46]. This would support the longer incubation time required for isolates from the faucet swabs, recovered after 96 to 240 h (Table 1). However, the absence of chlorine residuals and the presence of plastic aerators and warmer water temperatures within the faucet could provide favorable conditions for *Pa* growth. Therefore, depending on the specific conditions present at the faucet, the *Pa* positivity may vary between 7% and 74% as reported [12,22,47].

The positivity for sink surfaces was 12.5%, of which 50% had positive drains, 10% had positive faucets and 10% had both sites positive. Although the odds ratio for having a contaminated sink surface was 4.6 times higher when the faucet swab rather than drain was positive, the high prevalence of contaminated drains associated with sink surfaces suggested the drain as the primary reservoir of *Pa* contamination of the sink surface [26], especially if the disposal of patient body fluids or wastewater occurred in those drains [48]. On the other hand, the use of the shorter incubation time for the survey of hospital B to E could have impacted the positivity percentage for the faucet, the water and the aerator. When comparing positivity data for hospital A versus hospitals B to E, the drain positivity was lower (33% vs 51%), while the faucet and sink surface positivities were higher (17% and 22% vs 1% and 10%; Table 2). Considering that 84.5% of confirmed *Pa* strains isolated from the faucet or the sink surface required more than 48 h, the actual positivity for faucets and sink surfaces from hospitals B to E could have been up to 4.5 times higher than measured, especially considering the higher contamination rate detected in drains from hospitals B to E.

## 4. Materials and Methods

The culture method comparison was performed with environmental samples collected from a 255 bed adult hospital in the province of Québec, Canada (hospital A), supplied with chlorinated municipal water and no on-site disinfection. Swab samples were collected from sinks in rooms located in the following clinical and technical units: surgery, oncology, hemodialysis, emergency, geriatric, neonatology and hydrotherapy. Sterile cotton swabs (Puritan Medical Products) were used to collect biofilms from the drains, the tap aerators and the upper sink surfaces of 18 sinks, for a total of 57 samples, including 3 additional swab samples from a single sink drain. The samples were inoculated on PIa (DIFCO 292710) and Aa (Sigma-Aldrich 00185 with phenol red and agar) culture media. Incubation was performed at 35 °C for up to 240 h, and the subculturing of presumptive colonies took place after 24, 48, 96 and 240 h. Final confirmation was obtained through ISO 16266 standard culture method [49] and by gyrB qPCR [50]. In summary, colonies were plated on cetrimide agar with nalidixic acid (45.3 g/L cetrimide selective agar (Remel), 10 mL/L glycerol (Fisher), 15 mg/L nalidixic acid (Sigma-Aldrich)), incubated at 37.5 °C for 48 h and counted after 24 and 48 h. Following DNA chloroform extraction, the gyrB qPCR was performed by amplification using a Corbett Rotor-Gene 6000 for 50 cycles: a 10 min initial denaturation (95 °C), denaturation (95 °C; 30 s), annealing and elongation (60 °C; 90 s) [8].

MLVA genotyping was performed on 38 confirmed environmental isolates from sinks in hospital A and 32 clinical isolates from patients admitted throughout hospital A. Clinical isolates were uniformly distributed in time 24 months prior to and 6 months after the environmental sampling. DNA was extracted using a phenol method and the selected VNTR loci (MS142, MS211, MS213, MS215, MS216, MS222, MS223) were amplified by PCR using primers and conditions described by D. Sobral et al. [34]. The number of repeats for each VNTR locus were determined by electrophoresis on 2% agarose gel.

The occurrence study was conducted over 4 months, in five healthcare centers (identified as hospitals A to E) in the province of Quebec (Canada), including four adult hospitals (A: 255, C: 405, D: 420, and E: 80 beds) and one pediatric hospital (B: 450 beds). All hospitals were supplied with their municipal chlorinated water and no on-site treatment was used. A total of 229 sinks were sampled in the different study sites (18, 60, 52, 30 and 68, respectively). For each sink, the samples were collected as follows: (a) from a swab from the drain, (b) from 1 L of first-flush cold water in a sterile propylene bottle with 1% sodium thiosulfate (hospitals B to E), (c) from a swab of the aerator, and (d) from a swab of the upper sink surface (hospitals A and B). For water samples, 10 and 100 mL were filtered on a 0.45 μm cellulose membrane in duplicate. Filters and swabs were directly plated on the selected growth media using the ISO16266 method for hospitals B to E. Culture detection was performed on PIa and Aa selective media for samples from hospital A, with confirmation by ISO16266 and qPCR as described in the previous section.

The index of diversity (IOD) was calculated using the HGDI, using the V-Dice application (http://www.hpabioinformatics.org.uk/cgi-bin/DICI/DICI.pl). Statistical analyses were performed using the chi-square test, and were considered significantly different if *p* ≤ 0.05.

## 5. Conclusions

In this study, a large variety of environmental and clinical genotypes were isolated within multiple wards of a healthcare facility. Some genotypes were predominant within the hospital, recovered from drains, faucets and splash areas from multiple sinks. However, it was one of the non-dominant genotypes that was recovered both from the environment and from patients. In addition, the environmental strain was recovered after 72 h of incubation, and would have been missed with a 24–48 h incubation. A longer incubation time was shown to be critical for the recovery of environmental strains, especially for strains isolated from less favorable environments such as faucets. This increases the likelihood of finding environmental strains that are genotypically identical to clinical strains, as was the case here for genotype B.

The large proportion of positive drains and the association with positive faucets or splash areas confirm hospital drains as important *Pa* reservoir, firstly as a nutrient-rich environment compared to faucets or sink surfaces, and secondly as a source of contamination via aerosols toward surrounding surfaces (hands, bed, aerator, sink, and countertop). This is exacerbated in situations for which drainage is deficient, allowing water to accumulate in the sink during utilization. Strains present in the drain can then be resuspended in that water, increasing the likelihood of transmission. The redundancy of several genotypes observed in the sink environment, on the patient or on both suggests the presence of certain pathways bridging these sites. The results also provide some evidence that clinical strains can be recovered from sinks, but further studies are needed to substantiate this finding. Detailed prospective studies using optimized culture methods with a longer incubation time and genotyping are needed to better understand and document the strain dynamics in the environment surrounding hospitalized patients. Such understanding is key for infection prevention, as it will provide the necessary information to define, prioritize and implement corrective and preventive measures.

## Figures and Tables

**Table 1:** Phenotypically different environmental isolates from hospital A on acetamide agar (Aa) and *Pseudomonas* isolation agar (PIa) after incubation times of 48, 72, 96, and 240 h. Multiple strains could be isolated from a given sample.

	Confirmed/Presumptive *Pseudomonas aeruginosa (Pa)* Isolates						
	(Confirmed/Presumptive *Pa*-Positive Environmental Sample)						
Incubation Time (h)	Drain		Faucet		Surface		Total
	Aa	PIa	Aa	PIa	Aa	PIa	
48	9/13	12/12	—	—	0/1	2/4	23/30
	(7/8)	(7/7)	—	—	(0/1)	(2/2)	(10/12) ^1^
72	2/2	2/2	—	—	—	0/1	4/5
	(1/1)	(1/1)	—	—	—	(0/1)	(1/2) ^1^
96	—	—	—	3/4	1/1	—	4/5
	—	—	—	(1/2)	(1/1)	—	(2/3)
240	—	—	—	4/4	—	3/3	7/7
	—	—	—	(2/2)	—	(2/2)	(4/4)
Total	11/15	14/14	—	7/8	1/2	5/8	38/47
	(8/9)	(8/8)	—	(3/4)	(1/2)	(4/5)	(17/21)

^1^ If isolated on Aa and PIa from same sample, counted as one positive sample.

**Table 2:** Percentage of *Pseudomonas aeruginosa (Pa)*-positive sites for an incubation time of up to 240 h compared to an incubation time of 48 h.

Hospital	Incubation Time (h)	% *Pa*-Positive (*n* Sampled Sites)		
		Drain	Faucet	Surface
A	Up to 240	33 (18)	17 (18)	22 (18)
	48 ^1^	28 (18)	0 (18)	6 (18)
B to E	48 ^1^	51 (210)	1 (210)	10 (60)

^1^ *p* ≤ 0.05.

**Table 3:** Multiple-locus variable-number of tandem repeats analysis results: typability and Hunter-Gaston diversity index (HGDI) for environmental and clinical strains from hospital A.

	MS142	MS211	MS213	MS215	MS216	MS222	MS223
All strains (*n* = 70)							
No. of observed alleles	6	7	7	6	3	6	5
Alleles	1–5; 7	2–8	1–5; 8; 9	1–6	1–2; 4	1–6	2–5; 7
Typability (%)	96	89	90	86	96	82	86
HGDI	0.72	0.83	0.72	0.75	0.49	0.74	0.60
All environmental strains (*n* = 38)							
No. of observed alleles	4	4	3	4	2	2	3
Alleles	1–2; 4–5	2–4; 6	1; 4–5	1–2; 4; 6	1–2	2; 4	2–4
Typability (%)	100	97	100	100	95	84	92
All clinical strains (*n* = 32)							
No. of observed alleles	6	7	7	6	3	6	5
Alleles	1–5; 7	2–8	1–5; 8; 9	1–6	1–2; 4	1–6	2–5; 7
Typability (%)	91	79	79	70	97	79	79
Youenoue et al., 2014 (*n* = 62)							
No. of observed alleles	9	9	11	8	5	7	6
Alleles	1–7; 12	2–9	0–7; 9–10	1–7	1–5	1–5; 7	2–7
Typability (%)	100	97	98	97	98	99	100
HGDI	0.83	0.79	0.86	0.86	0.65	0.76	0.70
Vu-Thien et al., 2007 (*n* = 24)							
No. of observed alleles	9	8	7	7	4	7	7
Alleles	1–7	2–8	3–7; 9	1–6	1–4	1–6	2–7
Typability (%)	100	100	96	100	96	100	75
HGDI	0.81	0.76	0.85	0.80	0.64	0.76	0.77

**Table 4:** Percentage of paired positivity between two sampling sites at a sink for drains, faucets and sink surface swabs for hospitals A and B (*n* sink = 86) and for drain swabs, faucet swabs and water samples in hospitals B, C, D and E (*n* sink = 210). Odds ratios are indicated in brackets per sampling site pairs.

			**% Paired *Pseudomonas aeruginosa* Positivity at a Sink (Odds Ratio)**		
		***n*** ** Positive**	**Drain**	**Faucet**	**Sink Surface**
Hospitals A & B	Drain	108	—	7 (3.3)	20 (1.3)
	Faucet	3	67	—	40 (6)
	Sink surface	9	60	20	—
		***n*** ** Positive**	**Drain**	**Faucet**	**Water**
Hospitals B, C, D & E	Drain	108	—	7 (3.3)	20 (1.3)
	Faucet	3	67	—	40 (6)
	Water	9	60	20	—

## Supplementary Materials

The following are available online at www.mdpi.com/2076-0817/6/3/36/s1: Table S1: Genotype grouping of environmental (CL) and clinical (H) isolates from hospital A as per MLVA-7 profiles and location of isolation (environmental) or acquisition (clinical).
