# Supplementary material for: Hospital Drains as Reservoirs of Pseudomonas aeruginosa: Multiple-Locus Variable-Number of Tandem Repeats Analysis Genotypes Recovered from Faucets, Sink Surfaces and Patients
Source: Pathogens. 2017 Aug 9;6(3):36. doi: 10.3390/pathogens6030036 (PMC5617993; doi:10.3390/pathogens6030036)
Supplement: Supplementary file 1 [file pathogens-06-00036-s001.pdf]

TABLE S1 Genotype grouping of environmental (CL) and clinical (H) isolates from hospital A as per MLVA-7 profiles and location of isolation (environmental) or acquisition (clinical)

Genotype (nb of VNTR missing)	Strain ID	Location	MS142	MS211	MS213	MS215	MS216	MS222	MS223
A	H517	-	1	3	5	6	1	1	4
A	H519	-	1	3	5	6	1	1	4
A(-2)	H524	Nephrology	1	-	5	-	1	1	4
B	CL513	Geriatric	1	2	1	2	1	2	4
B	H511	-	1	2	1	2	1	2	4
B(-2)	H532	Geriatric	1	-	1	2	1	2	-
C(-1)	H501a	-	2	2	5	2	1	-	2
C(-1)	H503	-	2	2	5	2	1	-	2
C(-1)	H504	-	2	2	5	2	1	-	2
D	H514	-	4	7	5	5	1	4	7
D	H515	-	4	7	5	5	1	4	7
E	CL501	Oncology	4	6	4	1	2	2	2
E	CL502	Oncology	4	6	4	1	2	2	2
E	CL504	Oncology	4	6	4	1	2	2	2
E	CL505	Oncology	4	6	4	1	2	2	2
E	CL506b	Oncology	4	6	4	1	2	2	2
E	CL507	Oncology	4	6	4	1	2	2	2
E	CL508	Oncology	4	6	4	1	2	2	2
E	CL528	Oncology	4	6	4	1	2	2	2
E	CL529	Oncology	4	6	4	1	2	2	2
E	CL534a,b,x	Oncology	4	6	4	1	2	2	2
E	CL535	Oncology	4	6	4	1	2	2	2
E	CL542a,b	ICU	4	6	4	1	2	2	2
E	CL545a,b	Surgery	4	6	4	1	2	2	2
E	CL547a	Laundry	4	6	4	1	2	2	2
E	CL549	Laundry	4	6	4	1	2	2	2
F(-1)	CL511	Geriatric	5	4	5	1	2	-	2
F(-1)	CL512	Geriatric	5	4	5	1	2	-	2
F(-1)	CL519	Neonatology	5	4	5	1	2	-	2
F(-1)	CL520	Neonatology	5	4	5	1	2	-	2
F(-1)	CL532	ICU	5	4	5	1	2	-	2
G	CL515	Geriatric	5	3	5	6	2	4	2
G	CL516	Geriatric	5	3	5	6	2	4	2

TABLE S1 (continued)

Genotype (nb of VNTR missing)	Strain ID	Location	MS142	MS211	MS213	MS215	MS216	MS222	MS223
H	CL522b	ICU	5	4	5	4	2	2	3
H	CL524	ICU	5	4	5	4	2	2	3
H(-1)	CL521b	ICU	5	4	5	4	2	2	-
H(-1)	CL523b	ICU	5	4	5	4	2	2	-
ST01	H516	-	1	2	8	2	1	5	3
ST02	H520	Surgery	1	3	5	6	2	4	2
ST03(-1)	H529	-	1	4	2	-	1	2	3
ST04	H518	-	1	6	9	3	1	3	3
ST05	H507	-	3	8	5	1	2	1	2
ST07(-2)	H531	Surgery	3	-	8	4	2	4	-
ST08	H506	-	4	6	2	5	2	3	3
ST09	H505	-	4	8	4	1	2	3	5
ST10(-1)	CL547b	Laundry	4	-	4	1	2	2	3
ST11	H527	Oncology	4	3	3	6	2	3	2
ST12	H510	-	4	4	3	5	2	4	2
ST13	CL517	Neonatology	4	4	5	1	2	4	2
ST15(-1)	H512	-	4	3	5	1	2	6	-
ST16	H513	-	4	3	4	4	2	3	2
ST17	H502	-	4	3	4	2	2	2	2
ST18	H508	Oncology	4	2	3	6	2	2	2
ST19(-1)	CL509	Geriatric	2	4	5	1	2	-	2
ST20(-1)	H509		5	2	5	2	1	-	2
ST21(-1)	CL521c	ICU	5	4	4	4	2	2	-
ST22	CL527	Oncology	5	6	4	1	2	2	2
ST24(-2)	CL514	Geriatric	-	3	5	1	-	4	2
ST25(-2)	H525a,b	-	4	5	-	-	4	3	2
ST26	H522	Geriatric	7	7	3	4	1	4	3
ST27	H528	-	7	2	3	-	1	3	7
ST28	CL521a	-	5	4	5	1	0	4	2
Not classified	H521	-	4	-	-	-	2	-	2
Not classified	H523	-	-	-	-	-	-	-	-
Not classified	H526	ICU	-	-	-	-	1	-	-

Note: a,b,c,x represent strains isolated from the same swab sample. Results combined in the above table (n=66 strains) but kept separate for the statistical table (Table 2, n=71)
